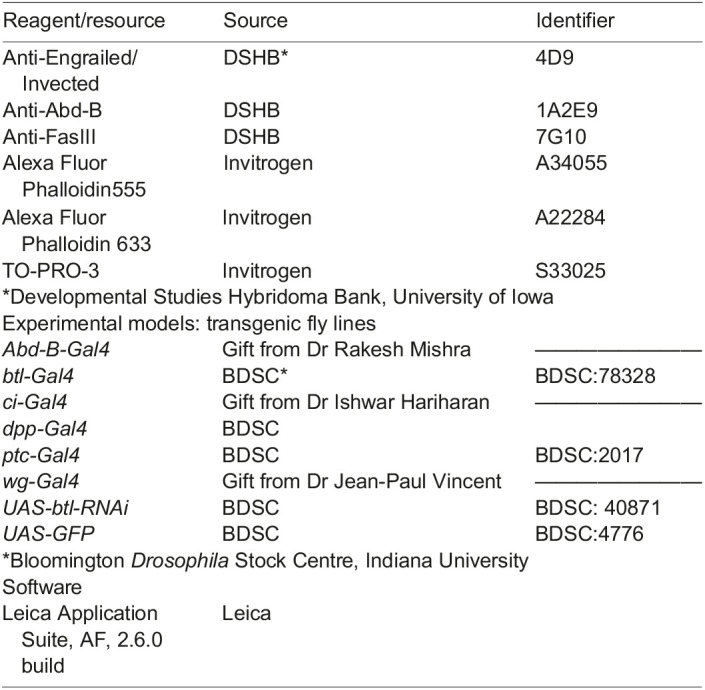# Developmental expression patterns of toolkit genes in male accessory gland of *Drosophila* parallels those of mammalian prostate

**DOI:** 10.1242/bio.058722

**Published:** 2021-08-17

**Authors:** Jaya Kumari, Pradip Sinha

**Affiliations:** Department of Biological Sciences and Bioengineering, Indian Institute of Technology Kanpur, Kanpur 208016, India

**Keywords:** *Drosophila*, Male accessory gland, Prostate, Developmental design

## Abstract

Conservation of genetic toolkits in disparate phyla may help reveal commonalities in organ designs transcending their extreme anatomical disparities. A male accessory sexual organ in mammals, the prostate, for instance, is anatomically disparate from its analogous, phylogenetically distant counterpart – the male accessory gland (MAG) – in insects like *Drosophila*. It has not been ascertained if the anatomically disparate *Drosophila* MAG shares developmental parallels with those of the mammalian prostate. Here we show that the development of *Drosophila* mesoderm-derived MAG entails recruitment of similar genetic toolkits of tubular organs like that seen in endoderm-derived mammalian prostate. For instance, like mammalian prostate, *Drosophila* MAG morphogenesis is marked by recruitment of fibroblast growth factor receptor (FGFR) – a signalling pathway often seen recruited for tubulogenesis – starting early during its adepithelial genesis. A specialisation of the individual domains of the developing MAG tube, on the other hand, is marked by the expression of a posterior Hox gene transcription factor, Abd-B, while Hh-Dpp signalling marks its growth. *Drosophila* MAG, therefore, reveals the developmental design of a unitary bud-derived tube that appears to have been co-opted for the development of male accessory sexual organs across distant phylogeny and embryonic lineages.

This article has an associated First Person interview with the first author of the paper.

## Abstract (precis)

**Summary:** We show genetic toolkit conservation between *Drosophila* MAG and mammalian prostate may suggest a common modular developmental design.

## INTRODUCTION

Conservation of genetic toolkits – the molecular architects of animal body plan – has helped reveal underlying developmental principles across a broad phylogenetic spectrum (Carroll et al., 2001; Shubin et al., 2009). Deconstructions of anatomical features into their independent or modular components often reveal conservation of genetic toolkits and thereby co-option of developmental designs via evolutionary lineages or independent innovations across distant phyla (Brandon, 2005; Rasskin-Gutman, 2005). For instance, respiratory organs in two disparate phyla: namely, endoderm-derived mammalian lungs (Swarr and Morrisey, 2015) and ectoderm-derived trachea in the invertebrate *Drosophila* (Ghabrial et al., 2003) recruit common toolkits: that is, FGF and Hh signalling (Butí et al., 2014; Chuang et al., 2003). Comparison of expression of shared genetic toolkits thus helps unravel modularity in the developmental design and ground plan of analogous organs from distinct developmental and phylogenetic lineages.

The mammalian prostate is derived from a common endodermal embryonic primordium of reproductive and urinary organs termed as urogenital sinus (UGS) (Marker et al., 2003; Shapiro et al., 2004). Specification of prostatic UGS at its pre-bud stage is followed by its budding, bud elongation, branching and, finally, canalization within each of its primary and secondary branches that create their lumens (for review, see Cunha et al., 2018). Epithelial linings of these lumens differentiate into two secretory cell types: luminal and basal (for review, see Marker et al., 2003). Cell fate specification of the prostatic primordium is marked by the gain of homeodomain transcription factors, such as Nkx3.1 (for review, see Prins and Putz, 2008), while recruitment of fibroblast growth factor (FGF) and sonic hedgehog (Shh) signalling regulate their growth and branching (Doles et al., 2006; Donjacour et al., 2003; Le et al., 2020; Lin et al., 2007). Posterior Hox genes: for instance, Hoxb13 induces cell differentiation in the luminal cell (Economides and Capecchi, 2003; Huang et al., 2007).

Prostate-like protein-rich seminal fluid secreting organs termed male accessory glands (MAG) are also present in invertebrates, as in the dipteran insect, the fruit fly, *Drosophila* (Findlay et al., 2008; Gilany et al., 2015; Ravi Ram and Wolfner, 2007; Verze et al., 2016). By contrast to the endodermal mammalian prostate, *Drosophila* MAG is mesodermal in origin and represents a paired tubular organ (Ahmad and Baker, 2002). It originates from an adepithelial primordium – which develops in close apposition with the ectodermal male genital disc epithelium – and gives rise to both MAG and seminal vesicle (SV). The *Drosophila* counterpart of mammalian fibroblast growth factor receptor (FGFR), Breathless (Btl), marks the common embryonic MAG-SV primordium (Ahmad and Baker, 2002). Adult MAG is a relatively simple tubular organ; its lumen is formed by squamous epithelium marked by two differentiated cell types: main and secondary, and is overlaid by circular rings of contractile muscles (Bairati, 1968; Susic-Jung et al., 2012).

The mammalian prostate is proposed to have appeared nearly 65 million years ago (Coffey, 2001), and its evolutionary origin seems to be independent of that of its *Drosophila* counterpart, MAG. Here we show that MAG development in *Drosophila* reveals an essential unitary prostatic tubule formed by shared genetic toolkits, recruited during mammalian prostate development. *Drosophila* MAG, therefore, displays a modular developmental ground plan, which appears to have been co-opted across distant phyla.

## RESULTS AND DISCUSSION

### Spatial coordinates of developing MAG-SV and genital primordia

**Fig. 1.**
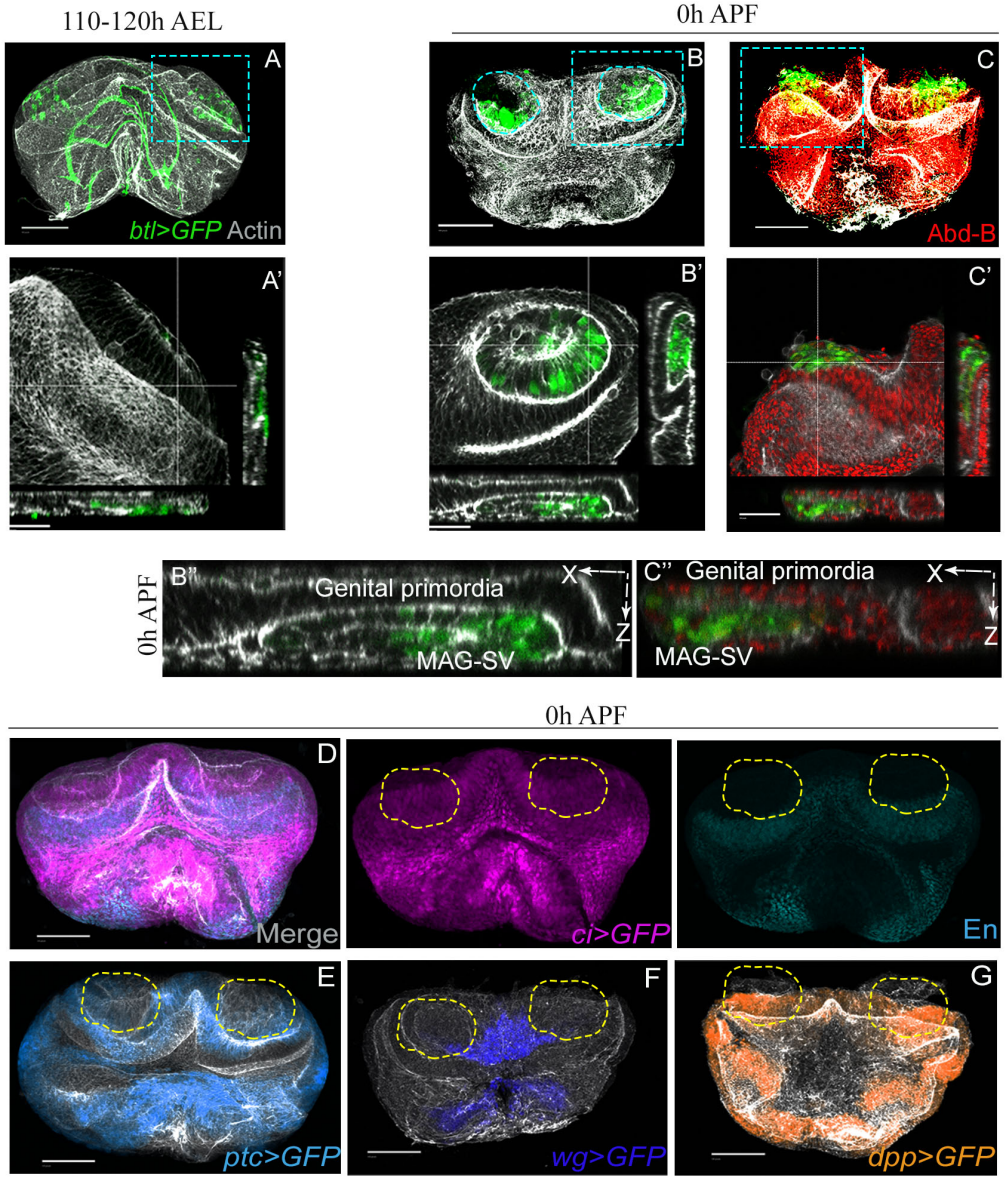
**MAG-SV and genital primordia develop in close apposition.** (A–C) GFP marked MAG-SV primordium in the backdrop of the genital imaginal disc at (A) third instar and (B,C) 0 h APF. Zoomed box A′ (Actin, grey) depicts remodelling of the imaginal disc at the third instar, while *btl-Gal4>UAS-GFP* marked MAG-SV primordial cells lodge. Zoomed boxes (B′) show the clustered MAG-SV primordium, (B″) show the apposition of genital and MAG-SV primordia in X-Z section, and (C′,C″) the characteristic large nuclei of MAG-SV primordial cells. X-Z view at 0 h APF in B′ and C′ is further enlarged below. (D–G) Genital disc patterning genes displayed by their pseudo-coloured GFP and immunostaining (En) depict the spatial position of overlaying MAG-SV primordium, which is denoted by broken lines. Scale bars: 100 µm. AEL, after egg laying; APF, after puparium formation.

In third instar male larvae, a group of mesodermal adepithelial cells of the genital disc – marked by expression of Btl/FGFR – migrate and lodge onto the epithelium of the genital imaginal disc. These adepithelial cells represent a common primordium of SV and MAG (Ahmad and Baker, 2002) (see Fig. 1A). The MAG-SV primordium thus develops in close apposition with the genital disc from the third larval instar till puparium formation (Fig. 1A–C). Optical cross-section further revealed that MAG-SV primordium is surrounded by the genital disc epithelium (Fig. 1A′–C′, zoomed cross-section in B″), the latter marked by a *Drosophila* posterior Hox gene *Abd-B* (Fig. 1C′, zoomed cross-section in C″) (de Navas et al., 2006; Estrada and Sánchez-Herrero, 2001). The MAG-SV primordium displays the expression of *engrailed* (*en*) and *cubitus interruptus* (*ci*) (Fig. 1D), marking its presumptive posterior and anterior compartments, respectively, wherein *patched* (*ptc*) (Fig. 1E) expression straddles their boundary while signalling pathways like Wingless (Wg) (Fig. 1F) and Decapentaplegic (Dpp) (Fig. 1G) regulate its morphogenesis (Casares et al., 1997; Chen and Baker, 1997; Freeland and Kuhn, 1996). The mesodermal MAG-SV and the ectodermal genital primordia thus develop in close apposition, prefiguring their mutual contacts in the adult.

### Spatio-temporal expressions of Btl/FGFR and Abd-B during MAG development

**Fig. 2.**
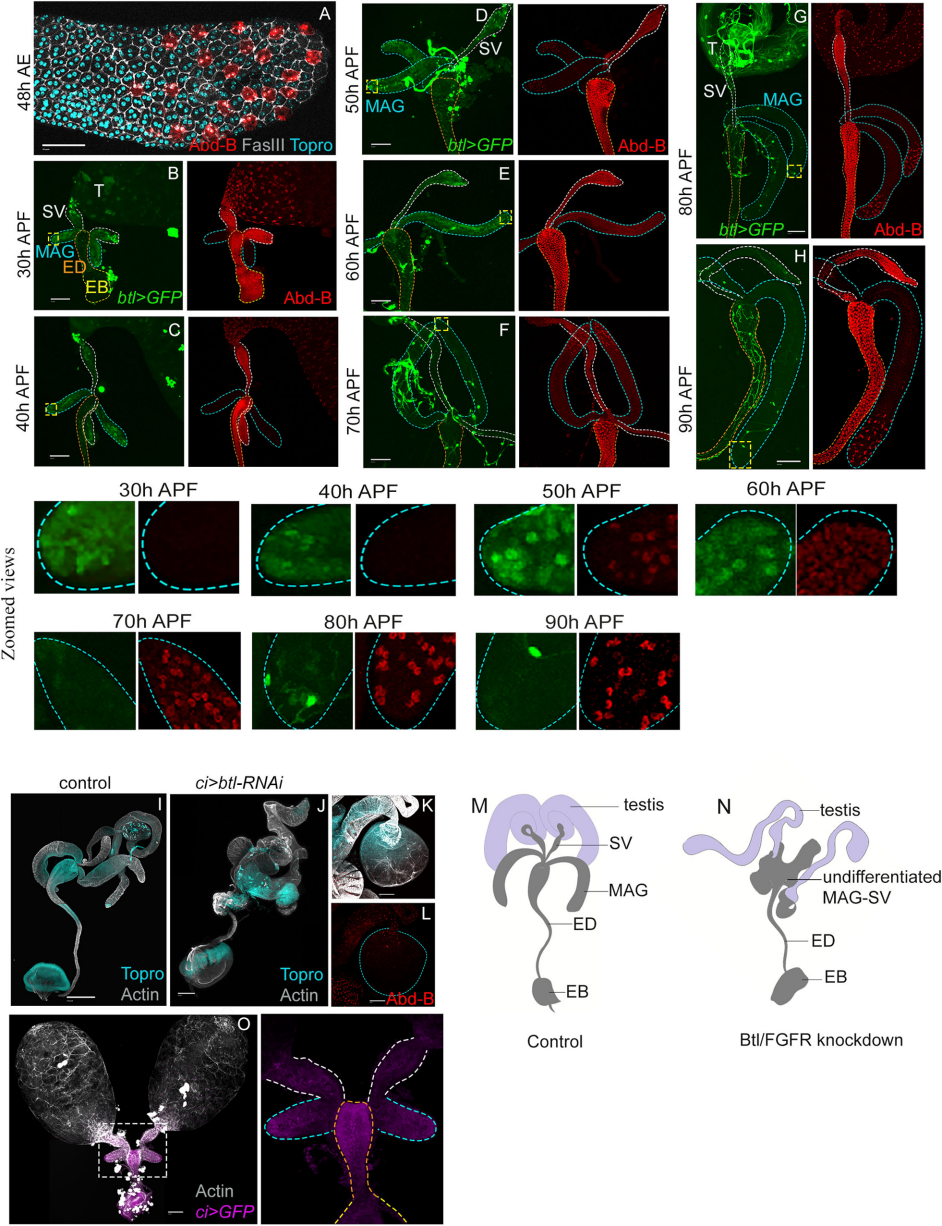
**Spatio-temporal expressions of Btl/FGFR and Abd-B during pupal MAG morphogenesis.** (A) Cell outlines of MAG epithelium (Fas III, grey) and cell nuclei (Topro, cyan) from a 2 day old adult after eclosion (AE), secondary cells are marked by Abd-B (*Abd-B>GFP*, red pseudocolour). (B–H) Expression pattern of Btl (*btl-Gal4>UAS-GFP*) and Abd-B (red) in the MAG-SV primordium derivates, MAG and SV, are shown from 30–90 h APF. Distal tips in each image are shown at higher magnification in the bottom panel. Bright threads in D–H are the trachea. Images in B–H are representative of five to eight samples examined at each time point. (I) Control *(ci-Gal4)* male reproductive system (*n*=20) compared to (J) knockdown of *btl (ci>btl-RNAi)*(*n*=20) during MAG development marked by Actin (grey) and Topro (cyan). (K–L) shows a sample truncated MAG (*n*=15/20) upon Btl knockdown, which displays loss of (L) Abd-B marked secondary cells in it. (M,N) Cartoon renditions of (M) control and (N) Btl knockdown are shown on the extreme right. (O) Expression of *ci-Gal4* driven UAS-GFP is shown in the male reproductive system at 30 h APF. The boxed area is zoomed onto the right. Scale bars: 100 µm. Testis (T); male accessory gland (MAG, broken blue line); seminal vesicle (SV, white dotted line); ejaculatory duct (ED, orange dotted line); ejaculatory bulb (EB, yellow dotted line).

Mammalian (rodent) prostate develops from multiple buds that spatially form bilaterally symmetrical anterior, dorsolateral, and ventral lobes (Lin et al., 2003; Marker et al., 2003; Timms, 2008). FGFR expression during prostate development is seen selectively during growth and branching morphogenesis; its expression being robust at the distal tips of these branching buds revealing its critical role as a toolkit signalling pathway during rodent prostate development (Huang et al., 2005). Thus, upon conditional knockdown of FGFR, the anterior and the ventral lobes are selectively lost while ductal patterning of the dorsolateral lobe compromised (Lin et al., 2007). In all prostatic lobes, the posterior Hox, Hoxb13 is expressed explicitly in the ductal epithelium, its highest expression being the luminal cells (Economides and Capecchi, 2003; Huang et al., 2007). MAG-SV primordium at late third instar, too, expresses Btl/FGFR (Ahmad and Baker, 2002). The sole *Drosophila* posterior Hox gene, *Abd-B* (Coiffier et al., 2008), on the other hand, is selectively expressed in only the differentiated secondary cells of the adult MAG (Gligorov et al., 2013) [48 h after eclosion (AE), Fig. 2A]. Further, FGF signalling acts as a driver of directional migration as can be seen from Btl/FGFR expressing mesodermal adepithelial cells visualised by an enhancer trap Gal4, while these lodge onto the genital disc at the 3rd instar stage to form the presumptive MAG-SV primordia (Ahmad and Baker, 2002). Secondary cells of the adult MAG, too, display expression of Btl/FGFR in a sub-set of its secreted extracellular vesicles called exosomes (Fan et al., 2020).

These parallels between mammalian prostate and *Drosophila* MAG, therefore, hinted at similarities in their developmental design. To further explore and elaborate on this design of the developing MAG, we examined Btl (*btl-Gal4>UAS-GFP*) and Abd-B during the entire course of pupal morphogenesis of MAG. We noticed that Btl/FGFR robustly marks the MAG bud early during its morphogenesis (30–50 h APF, Fig. 2B–D). Further, around 50 h APF, Btl/FGFR expression turns robust in a group of distal cells (Fig. 2D, see zoomed distal tip 50 h APF), which is extinguished, shortly ahead of the conclusion of pupal development (Fig. 2E–H, 60–90 h APF). Therefore, this dynamic pattern of Btl/FGFR expression suggests its role during MAG bud growth and morphogenesis rather than in the adult gland. Further, such Gal4 lines may also fail to recapitulate the entire spectrum of Btl/FGFR activity in the MAG (see Fan et al., 2020).

The cell fate determinant posterior *Drosophila* Hox, Abd-B was not expressed in the growing MAG bud (30–50 h APF, Fig. 2B–D), unlike its expression in the developing SV throughout its budding and growth phase (Fig. 2B–H). Abd-B first appeared in the presumptive secondary cells of the MAG at 50 h AFP (Fig. 2D, see zoomed distal tip at 50 h APF). Interestingly, Abd-B, too, is initially expressed in the MAG in its main cells at 60 h APF (Fig. 2D, see zoomed distal tip at 60 h APF) but persists only in the secondary cells through the subsequent stages (Fig. 2D–H, also see zoomed distal tips). Therefore, Abd-B expression in developing MAG is marked by two characteristics: it's transient but ubiquitous expression in the entire gland, followed by its restricted expression in only the secondary cells.

Taken together, Btl/FGFR appears to be a primary toolkit for MAG-SV bud growth – a possibility that was further strengthened by the observation that its knockdown (*ci-Gal4>btl-RNAi*) truncates MAG growth (Fig. 2J,K) and induces loss of its cell differentiation marker for instance, by loss of the Abd-B-expressing secondary cells (Fig. 2L). We further recognise that, *ci-Gal4* expression (Fig. 2O) spans the nascent ejaculatory duct (ED), SV and proximal junction of SV and testis. Thus, the developmental fallout of *btl-RNAi* expression under the *ci-Gal4* driver may be due to Btl/FGFR loss in multiple organ primordia associated with the MAG besides some possible off-target effects of the RNAi. This caveat in the interpretation of our results notwithstanding, the fallouts of Btl/FGFR knockdown in the MAG are reminiscent of those seen in prostate precursor cells and their perturbed cell fate specifications (Lin et al., 2007).

Btl/FGFR signalling and posterior Hox toolkit expression pattern in the highly branched and multi-lobular mammalian prostate (Huang et al., 2007) therefore appears to be a reiterative deployment of a standard modular developmental design, as seen in *Drosophila* MAG.

### Hedgehog-Dpp signalling axis in MAG morphogenesis

In mouse prostate development, Shh signalling activity appears at budding; its expression declines thereafter and persists in the adult gland at very low levels (Doles et al., 2006; Lamm et al., 2002). The Shh ligand is seen enriched at the distal prostatic tips of the epithelial bud, the receptor (Ptc), transcription factor (Gli) and downstream targets (e.g. Bmp4) are prominently expressed in the surrounding UGS mesenchyme (Lamm et al., 2001, 2002; Podlasek et al., 1999; Pu et al., 2004). The developmental design reflected by Shh signalling for prostate development, however, remains ambiguous owing in part to genetic redundancy: for instance, Gli1 and Gli3 may compensate for Gli2 loss in UGS tissues (Doles et al., 2006). It has, however, not been ascertained yet if MAG displays the expression of the Hh signalling toolkit, reminiscent of that seen in the prostate. In the adult secondary cells, a downstream target of Hh, Dpp signal is active as suggested by the expression of its receptors Thickveins and Wishful Thinking (Redhai et al., 2016). Indeed, Dpp signalling regulates the shape, growth, and shedding of these cells in the adult gland (Leiblich et al., 2012; Redhai et al., 2016).

**Fig. 3.**
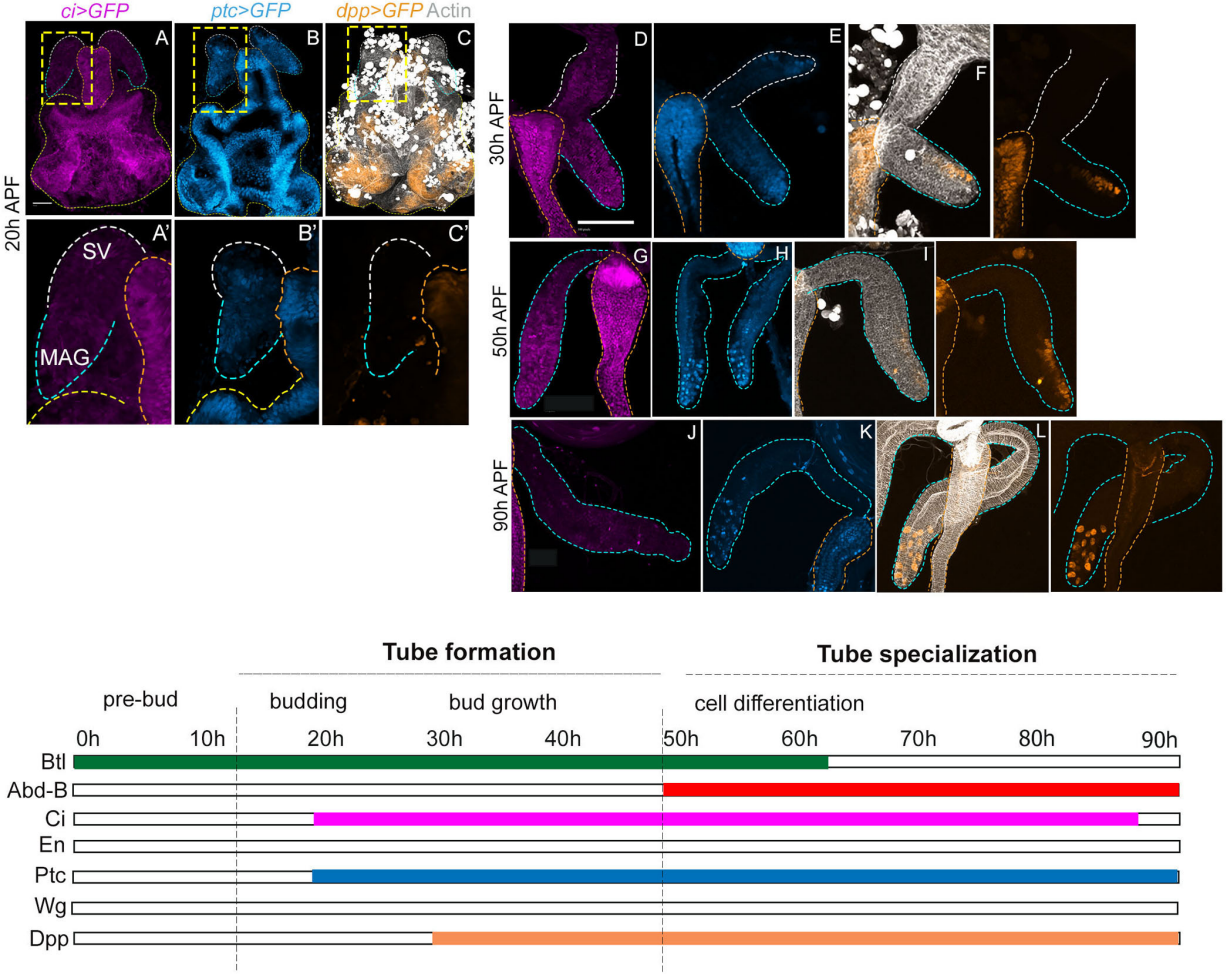
**Hedgehog-Dpp signalling axis during MAG morphogenesis.** (A–L) Expression of *ci* (A,D,G,J, purple), *ptc* (B,E,H,K, cyan blue) and *dpp* (C,F,I,L, orange) visualised by expressions of respective Gal4 driven UAS-GFP transgenes during budding (20 h APF, boxed areas zoomed below), bud growth (30 h APF) and cell differentiation (50 and 90 h APF) stages. Images in A–L are representative of five to seven samples examined at the denoted time point. The diagram below summarises the model of the MAG developmental ground plan based on its toolkit expressions. Btl/FGFR is a hallmark of the tube formation and Abd-B of tube specialization stage, while the Hh-Dpp signalling toolkit is common to both stages. Scale bars: 100 µm. Marking scheme of the individual organs are as in preceding Fig. 2.

We thus examined the expression of Hh pathway members in the MAG bud; Ci (*ci-Gal4>UAS-GFP*), Ptc (*ptc-Gal4>UAS-GFP*) and Dpp (*dpp-Gal4>UAS-GFP*) – a Hh target, and mammalian Bmp counterpart – during its pupal morphogenesis. While *ci>GFP* expression may not represent its actual activation – denoted by its cleaved form Ci-75 (Huangfu and Anderson, 2006; Price and Kalderon, 1999) – we consider it relevant for our purpose. We noted Ci expression around 20 h APF: that is, during budding of MAG (Fig. 3A, see zoomed box, A′). Further, Ci expression continues through its bud growth (Fig. 3D, 30 h APF) and cell differentiation stages (Fig. 3G, 50 h APF) before being finally extinguished (Fig. 3J, 90 h APF). The spatio-temporal expressions of Hh receptor Ptc, too, marked the budding (20 h APF, Fig. 3B zoomed box), bud growth (30 h APF, Fig. 3E) and cell differentiation stages (50 h APF, Fig. 3H), albeit more prominently at the distal end compared to that of Ci. Moreover, unlike Ci that extinguishes by the end of pupal development, Ptc was retained in the secondary cells (90 h APF, Fig. 3K), suggesting its further roles in these cells in the adult gland. Hh target, Dpp, however, did not mark the nascent bud (20 h APF, zoomed bud Fig. 3C). It faintly appeared at the distal end during bud growth (30 h APF, Fig. 3F) and cell differentiation (50 h APF, Fig. 3I). Its robust expression was noted in the secondary cells by the completion of pupal MAG morphogenesis (90 h APF, Fig. 3L).

These expression patterns during MAG development, therefore, suggest Hh signalling requirement during the bud growth stage and cell fate specification, although Btl/FGFR may be the earliest essential toolkit. Admittedly, our study based on Gal4 driver-driven GFP reporter expressions may not recapitulate the entire spectrum of Hh endogenous regulation. Nevertheless, relevant to the key question posed here, our study reveals uncanny parallels between Hh expression patterns in MAG development with those of Shh during mammalian prostate developmental on the following counts: its appearance in the nascent bud stage, followed by distal enrichment as the tube grows and, finally, a decline in its expression upon formation of the tube.

### Conclusion

**Fig. 4.**
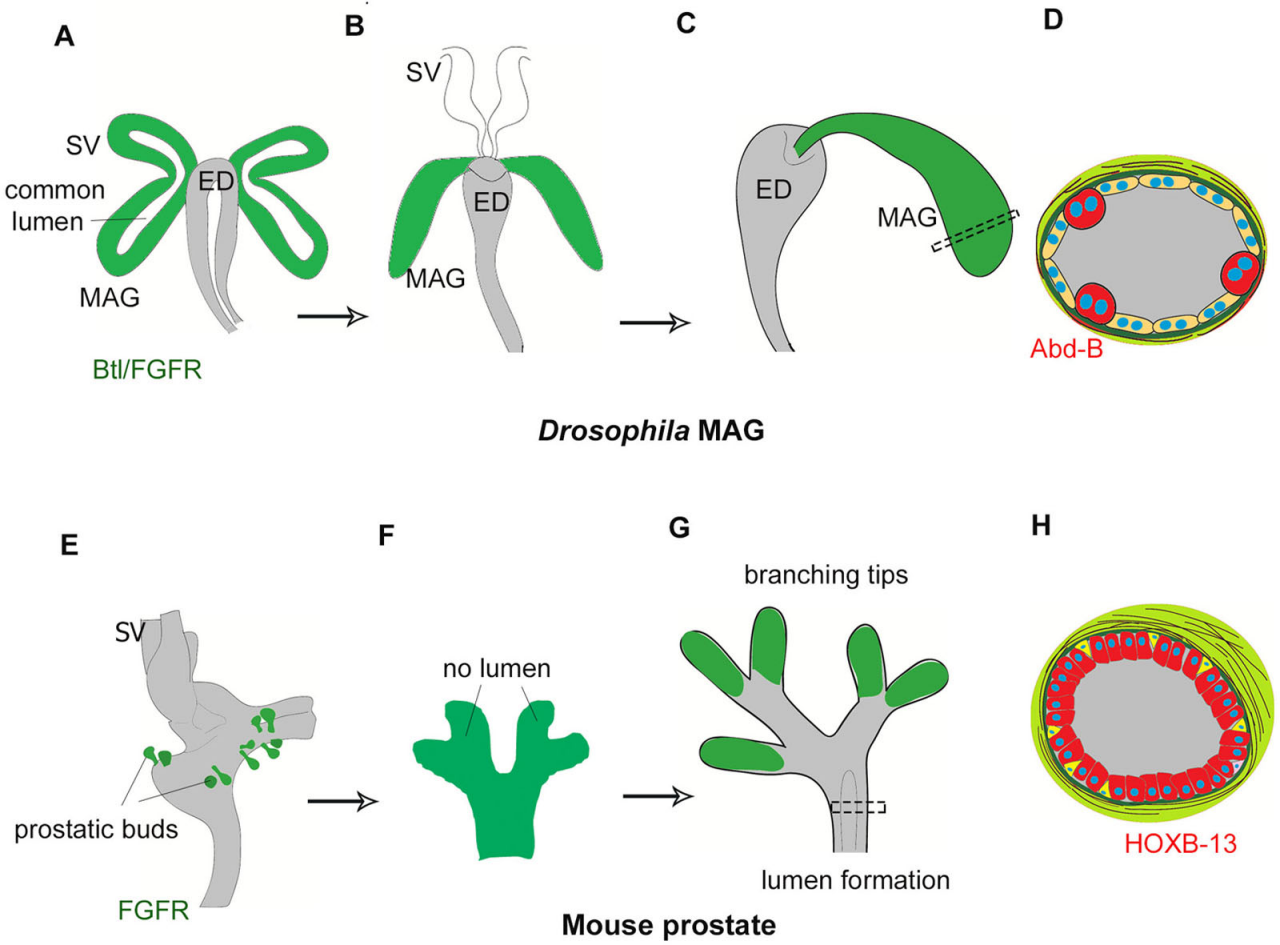
**Common developmental design of *Drosophila* MAG and mammalian prostate.** (A–C) Cartoon representation of MAG development from Btl/FGFR primordial cells: (A) unitary MAG and SV buds with *a priori* lumen appear from the MAG-SV primordium. (B) MAG grows as a short tube. (C) MAG eventually connects caudally with the ejaculatory duct. (D) Illustration of MAG cross-section with two differentiated cell types, secondary cells are marked by Abd-B. (E–G) Cartoon representation of mouse prostate development: (E) prostatic buds and SV are separate (F) individual primary prostatic bud does not have a lumen (G) branching with each individual tips marked by FGFR, while canalization precedes to form their lumen. (H) Illustration of prostatic cross-section depicting two differentiated cell types, luminal cells are marked by Hoxb13.

Our study reveals that the essential developmental design of MAG as a simplified tube recapitulates that seen in a mammalian prostatic bud (Fig. 4). The recruitment of shared toolkit genes also suggests co-options of common developmental ground plans (Shubin et al., 1997, 2009). We also note that Btl/FGFR and Hh/Shh signalling pathways are genetic toolkits for other tubular organs such as the *Drosophila* trachea and mammalian lungs (Hayashi and Kondo, 2018; Warburton et al., 2000). Thus, in a blind watchmaker's (Dawkins, 1986) evolutionary paradigm, common genetic toolkits are co-opted in distant phylogenies and in multiple organ types, thereby recreating comparable ground plans in analogous organs (Wagner, 2007; Wagner and Altenberg, 1996). These commonalities in ground plan notwithstanding, *Drosophila* MAG and mammalian prostate display disparate cellular features and tissue architecture: for instance, binucleation and cuboidal-to-squamous transitions in the former (Taniguchi et al., 2014; Wilson et al., 2017) and columnar luminal cells besides the appearance of progenitor neuroendocrine cells in the latter (Marker et al., 2003; Toivanen and Shen, 2017). These peculiarities of the secondary sexual organs in two distant phyla – besides their origins from two separate germ layers – therefore call for caution in extrapolating results from *Drosophila* MAG as a surrogate for human prostatic diseases such as cancers (Rambur et al., 2020).

## MATERIALS AND METHODS

### Selection of Gal4 drivers

Gal4 lines were selected to recapitulate endogenous expression patterns during embryonic development or in larval tissues. *btl-Gal4* Gal4 on the third chromosome is a transgenic construct containing the *btl* promoter that recapitulates Btl expression in the tracheal cells (Shiga et al., 1996). *ci-Gal4* on the second chromosome is a transgenic construct containing the *ci* promoter, which encompasses its transcription and translation start sites and recapitulates its embryonic expression pattern (Croker et al., 2006). *dpp-Gal4* on the third chromosome recapitulates endogenous Dpp expression pattern in the wing imaginal disc (Bosch et al., 2016; Masucci et al., 1990). *wg-Gal4* is a transgenic line containing 5 kb of regulatory sequence and recapitulates endogenous Wg expression pattern during embryonic development and in the imaginal discs (Pfeiffer et al., 2000). *ptc-Gal4* is an enhancer trap line carrying an insertion on the second chromosome that recapitulates the expression of Ptc in imaginal discs (Bosch et al., 2016; Hinz et al., 1994; Speicher et al., 1994). *Abd-B Gal4* (*Abd-B^LDN^Gal4*) recapitulates the expression pattern of Abd-B M isoform, while its antibody marks the entire Abd-B expression domain (de Navas et al., 2006).

### Collection and staging of pupal samples

Male pupae were identified by their prominent ovoid gonads. Males of the required genotype were selected by examining GFP expression under a fluorescent microscope. The 0 h was decided as previously described (Bainbridge and Bownes, 1981). The maximum time variation may range ±30 min amongst individuals of a given stage. Collected 0 h samples were allowed to grow at 25±1°C in a petri-dish with moist filter paper until the time of dissection. At least five individuals of a genotype were dissected and imaged for a given time point in two experimental replicates.

### Dissection of pupal MAG samples

Pupal dissection for 0–20 h APF samples was done by excising the lower third of the pupae using dissection scissors in cold PBS. The genital disc was gently pushed out of the pupal cover using insulin syringes. For 30–70 h pupal samples, first, a cut was made at the abdomen-thorax junction. The reproductive structures within the abdomen were gently pushed out using insulin syringes. The tissue was cleared of abdominal fat and lipids. The pharate stage animals between 80–90 h APF were taken out of the pupal case. MAG was isolated by gently removing the external genitalia using a pair of insulin syringes similar to adult dissection.

### Immunofluorescence staining and microscopy

In brief, samples were fixed in 4% paraformaldehyde for 35 min, washed with PBS and incubated with the desired primary antibody overnight. The samples were washed, blocked in bovine serum albumin for an hour before adding a secondary antibody. Samples were counterstained with TO-PRO-3 (Invitrogen) and/or Phalloidin. Primary antibodies used: Mouse anti-En (1:50), Mouse anti-Abd-B (1:50), Mouse anti-FasIII (1:100); Phalloidin 555, 633 (1:100), TOPRO (1:500). Finally, the prepared samples were mounted in Vectashield or Prolong Gold for imaging. Images were acquired using a Leica confocal SP5 system and processed using LAS AF software and Adobe Photoshop.

**Key resources table:**

**Table:**